# Comparison of Current International Guidelines for the Management of Alopecia Areata—Comprehensive Review

**DOI:** 10.3390/ijms26178632

**Published:** 2025-09-04

**Authors:** Julia Kropidłowska, Alexandra Kvinen, Miłosz Lewandowski, Roman J. Nowicki, Wioletta Barańska-Rybak

**Affiliations:** 1 Department of Dermatology, Venereology and Allergology, Faculty of Medicine, Medical University of Gdansk, Smoluchowskiego 17, 80-214 Gdansk, Polanda.kvinen@gumed.edu.pl (A.K.); milosz.lewandowski@gumed.edu.pl (M.L.); rnowicki@gumed.edu.pl (R.J.N.); 2 Practical and Experimental Dermatology Student Scientific Association, Faculty of Medicine, Medical University of Gdansk, Smoluchowskiego 17, 80-214 Gdansk, Poland

**Keywords:** general dermatology, medical dermatology, alopecia areata, guidelines, review

## Abstract

Alopecia areata is a persistent autoimmune-mediated disease with a complicated pathophysiology and a prevalence of approximately 2%. The exact pathogenesis is yet to be identified; nevertheless, environmental factors, autoimmune mechanisms and genetic factors among others all contribute to the multifactorial etiopathogenesis of the disease. Even though alopecia areata is frequently self-limiting and recovery can occur on its own, it can cause esthetic challenges that might precipitate psychosocial disorders. This article aims to provide a clinical update on alopecia areata comparing the most important international guidelines, with particular emphasis on current treatment options and comorbidities.

## 1. Introduction

Alopecia areata (AA) is a chronic autoimmune disorder characterized by the loss of immune privilege in anagen-phase hair follicles, leading to patchy areas of hair loss. Although it primarily affects the scalp, it may also involve the nails, eyelashes, eyebrows, and other hair-bearing sites across the body. The disease typically follows a relapsing-emitting course, often resulting in a substantial reduction in patients’ quality of life [1,2]. While the clinical manifestations of AA are well-known, the exact mechanism and etiology remain largely unclear. This article aims to provide a clinical update on alopecia areata, comparing the most important international guidelines, with particular emphasis on current treatment options and comorbidities [3,4,5,6,7].

## 2. Materials and Methods

This work is a narrative review comparing consensus guidelines for the treatment of alopecia areata from Europe, Australia, Brazil, Saudi Arabia, and international expert panels. We conducted systematic searches in the PubMed and Scopus databases for publications up to November 2024 using the query: “alopecia areata” AND (“treatment guidelines” OR “consensus” OR “management” OR “expert consensus”). The search yielded 249 records from PubMed and 81 records from Scopus, amounting to 330 articles in total.

**Figure 1:**
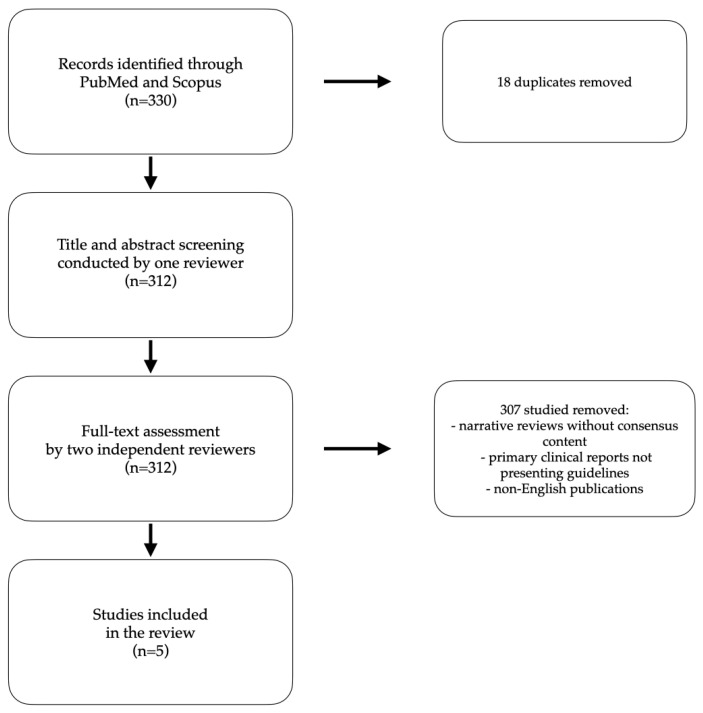
A summary of the identification, deduplication, and screening process presented in the flow diagram.

Inclusion criteria for articles were (1) the presentation of national or international consensus or guideline recommendations on alopecia areata treatment; (2) written in English; and (3) full-text availability. We excluded duplicates across databases, narrative reviews without consensus content, primary clinical reports not presenting guidelines, records not focused on AA management consensus, and non-English publications. The deduplication procedure was performed in Zotero. We identified and removed 18 duplicate records, resulting in 312 unique articles for further screening, the slightly lower figure observed in our review likely reflects the narrow focus of the search and database overlap characteristics. Following deduplication, title and abstract screening was conducted by one reviewer (A.K.), and full-text assessment by two independent reviewers (A.K.; J.K.). Any discrepancies were resolved through discussion, thereby minimizing bias and ensuring reproducibility (Figure 1).

## 3. Epidemiology

The estimated global prevalence of AA of approximately 2% underscores the importance of increasing awareness of this immune-mediated condition [8,9]. The disease can occur at any age, but its prevalence appears to be higher in children (1.92%) than in adults (1.46%) [9]. There seems to be no significant predilection to sex with a mean age of onset at 36 years of age in females and 32 years of age in males [10].

## 4. Diagnosis

The diagnosis of AA is primarily established on the basis of clinical features and trichoscopic findings. The most characteristic trichoscopic features include yellow dots, short vellus hairs, black dots, broken hairs, and exclamation mark hairs. Importantly, the diagnosis should rely on a combination of trichoscopic features rather than a single finding [11]. Rarely when the diagnosis remains uncertain, a punch biopsy is advisable. The histopathological features differ depending on the stage of the disease [12]. The most characteristic histopathological feature in the acute stage is a peribulbar lymphocytic infiltrate, composed predominantly of CD8^+^ and CD4^+^ T cells, often described as resembling “a swarm of bees.” Beyond the acute stage, this finding is not consistently observed. In such cases, the presence of lymphocytes (94%), melanin deposits (84%), and eosinophils (44%) within fibrous tracts may aid in establishing the correct diagnosis [13,14]. Furthermore, nail pitting and trachyonychia are common manifestations of the disease, occurring in approximately 7% to 66% of cases [15].

## 5. Pathogenesis

Pathogenesis of alopecia areata involves a complex interplay of genetic, immunologic and environmental factors, each contributing to disease onset, progression, and variability in presentation.

### 5.1. Genetics

Family-based linkage analyses, twin studies (both monozygotic and dizygotic), and investigations of first-degree relatives consistently demonstrate a strong genetic predisposition to AA, as reflected by the frequent occurrence of affected family members. In a study by Blaumeiser et al. (n = 206), 20% of patients had a first-degree relative with AA [16]. In a separate study by Rodriguez et al., which focused on twin patients, the concordance of AA was higher in monozygotic twins (42%) than in dizygotic twins (10%), drawing a positive correlation between AA and genetic factors [17].

### 5.2. Immunology

Immunological mechanisms are pivotal in the disease’s pathogenesis. The disruption of hair follicle immune privilege was first suggested by Paus et al. as the primary cause of AA [18]. The presence of lymphocytes, dendritic cells, and NK cells in the peribulbar region of anagen hair follicles indicates a collapse of immune privilege, as this area is normally devoid of immune cells. This collapse results in an autoimmune response against hair follicle autoantigens, ultimately leading to hair loss. Several studies have identified interferon-γ (IFN-γ) as a key contributor to this process [19]. The presence and functionality of regulatory T (Treg) lymphocytes represent another key mechanism in the pathogenesis of AA. Treg cells play a pivotal role in maintaining peripheral tolerance and preventing the development of autoimmune disorders, and they are particularly abundant within hair follicles [20]. Ali et al. demonstrated that Tregs in hair follicles aid in regeneration by boosting the quantity and diversity of stem cells [21].

### 5.3. Allergy

Allergy is hypothesized to be one of the factors in AA pathogenesis contributing to its onset, relapse, and severity. It has been proven that atopy increases the risk of developing AA [22]. Several studies reported increased serum IgE level and presence of eosinophils and mast cells in the lesions of AA patients [23,24,25]. Recurrent relapse and recovery is observed in AA patients, which could be a result of seasonal changes in allergen exposure [26]. A study by Li et al. showed a correlation between allergy to dust mites and severity of AA, as well as the condition’s possible early onset [27]. Antihistamines or desensitization for house dust mites could decrease severity of alopecia in atopic AA patients [28]. Additionally, Uchida et al. presented a case of a 44-year-old male with a past medical history of atopic dermatitis and recalcitrant alopecia areata. After 3 months of therapy with dupilumab in addition to a topical corticosteroid, the patient showed both a notable decrease in patient skin manifestations, as well as remarkable hair regrowth [28]. Dupilumab, a monoclonal antibody and an antagonist to IL-4 and IL-13 commonly used for allergic diseases, could be beneficial to AA patients with comorbid atopic dermatitis [28].

### 5.4. Oxidative Stress (OS)

Oxidative stress (OS) is defined as an excessive accumulation of reactive oxygen species (ROS) that arises when the body’s antioxidant defense mechanisms fail to adequately eliminate them, resulting in an imbalance between oxidation and reduction processes [29]. According to current evidence, there is a link between AA and ROS [30]. Sun Chao et al. conducted a study where the aim was to analyze and identify the key markers of OS in AA and vitiligo. The authors concluded that KLB and EIF3C are key genes in OS regulation of AA and vitiligo and that KLB and EIF3C participate in disease progression by regulating T cells and neutrophils [29]. Moreover, a recent meta-analysis demonstrated that patients with AA exhibit elevated serum levels of malondialdehyde, nitric oxide, and total oxidant capacity, along with reduced levels of superoxide dismutase, paraoxonase, glutathione peroxidase, and total antioxidant capacity. These findings suggest that further studies are warranted to investigate the potential therapeutic impact of modulating oxidative stress markers in AA [29,30].

### 5.5. Microbiota

The healthy hair follicle microbiota inhibits pathogen growth, promotes the synthesis of cytokines essential for initiating and sustaining immune responses, reduces inflammation, and supports tissue repair [31,32]. Microbiological studies of scalp biopsies performed by Pinto et al., discovered an increased number of *Anaerococcus* at the level of the epidermis in patients with AA [33]. *Anaerococcus* may play a role in the pathophysiology of AA as it has been shown to stimulate the release of adenosine monophosphate by keratinocytes in other inflammatory skin conditions [34]. In addition to Anaerococcus, elevated populations of *Neisseria* and *Acinetobacter* spp. have been observed in patients with AA, showing negative correlations with the FAS and SOD2 genes and a positive correlation with the NOD2 gene. These findings suggest a close interaction between the host and the microbiota in individuals with AA [35].

## 6. Comorbidities

Alopecia areata is frequently associated with various comorbid conditions, reflecting its complex autoimmune background. Understanding these comorbidities is crucial, as they may influence disease course, patient quality of life, and therapeutic decision-making Table 1.

## 7. Treatment

Numerous guidelines outline various treatment strategies for AA, consistently emphasizing the importance of a multidisciplinary approach to achieve effective disease management. Treatment selection is largely determined by clinical severity, which is commonly assessed using the Severity of Alopecia Tool (SALT). This tool quantifies scalp hair loss as a percentage, ranging from no hair loss (score 0) to complete baldness (score 100) [67]. The SALT score was developed to provide clinicians with a standardized method of assessing hair regrowth after therapy and is frequently employed in clinical trials to evaluate both disease severity and treatment effectiveness. However, it is limited to scalp hair only, and thus, a new scale has been proposed. The Alopecia Areata Scale (AAS) considers additional factors related to the disease’s severity beyond just the SALT score such as prior response to treatment or psychosocial impact of the disease [67,68].

Below is a comparative overview of AA treatment options based on current European, Australian, Brazilian, Saudi Arabian, and expert consensus guidelines [3,4,5,6,7]. Each article offers a varied perspective on the assessment and treatment of AA.

The European Expert Consensus considers a SALT score equal or greater than 20 a general indication for systemic therapy [3]. In contrast, the Australian Expert Consensus states that there are no universally agreed indications for initiating systemic treatment for AA and no evidence-based Australian or International treatment guidelines for systemic therapy of AA [6].

According to the Australian Expert Consensus, no systemic agents are currently approved by the Food and Drug Administration or the Therapeutic Goods Administration for the treatment of AA. This stands in marked contrast to the European Expert Consensus, where baricitinib (a JAK 1/2 inhibitor) is approved for adults and ritlecitinib (a JAK 3 inhibitor) is approved for both adults and adolescents with severe AA by the EMA. Both the European and Australian consensuses, however, recommend the off-label use of systemic agents such as glucocorticosteroids, cyclosporine, methotrexate, and azathioprine as potential treatment options [3,6].

While the Australian Expert Consensus defines treatment success as achieving a SALT50, the European Expert Consensus has more recently adopted SALT20 as the therapeutic goal. In addition, the Saudi Expert Consensus specifies treatment success as achieving any of the following: a 75% reduction in SALT score, a SALT score of 20 or less, or a Dermatology Life Quality Index (DLQI) of 5 or less [7].

### 7.1. Glucocorticosteroids (GCS)

According to the Alopecia Areata Consensus of Experts (ACE) study, topical corticosteroids may be used as first-line therapy, either as monotherapy or in combination, for AA of the scalp, eyebrows, or beard. For scalp involvement, a potent topical corticosteroid should be applied daily for at least 6 to 12 weeks and up to 3 to 6 months. However, potent topical corticosteroids should not be used on the eyelashes. For AA affecting the eyelashes, the ACE study recommends prostaglandin analogs, specifically latanoprost and bimatoprost, as first-line treatment, although neither the study nor current expert consensuses from Europe, Australia, Brazil, or Saudi Arabia specify the exact dosage formulations.

Furthermore, the ACE study reports that intralesional glucocorticoids are more effective than ultrapotent or potent topical corticosteroids in inducing hair regrowth and sustaining remission. The recommended regimen is triamcinolone acetonide at a concentration of 2.5–5 mg/mL, with a maximum of 10 mg/mL, applied cautiously near the frontal hairline due to the increased risk of atrophy. The Brazilian Expert Consensus suggests slightly broader dosing ranges, recommending 2.5–10 mg/mL of triamcinolone acetonide for the scalp and 2.5–5 mg/mL for the face and other body sites. Additionally, the Brazilian guidelines list betamethasone dipropionate (5 mg/mL), betamethasone disodium phosphate (2 mg/mL), and hydrocortisone acetate (25 mg/mL) as alternative intralesional treatment options [3,4,5,6,7].

Systemic glucocorticosteroids are widely used in the management of AA; however, there is no expert consensus regarding the optimal regimen for oral prednisolone. The ACE study advocates daily dosing of prednisolone or prednisone, recommending an initial dose of 0.4–0.6 mg/kg/day tapered gradually over more than 12 weeks to achieve sustained remission. In contrast, the Brazilian Expert Consensus suggests prednisone at doses ranging from 0.1 to 1 mg/kg/day, with an emphasis on initiating treatment at higher doses (0.5–1 mg/kg/day) followed by gradual tapering over 6–12 weeks once hair regrowth is achieved. Australian and Saudi experts recommend starting doses of 0.5–0.75 mg/kg/day and 0.4–0.6 mg/kg/day, respectively. Additionally, both European and Australian experts propose that pulse therapy with glucocorticosteroids may be considered, such as dexamethasone administered at 0.1 mg/kg/day on two consecutive days per week for several months [3,4,5,6,7].

### 7.2. Contact Immunotherapy

The ACE study recommends offering contact immunotherapy to children with alopecia universalis, alopecia totalis, or ophiasis before considering systemic therapy, emphasizing that treatment should be discontinued only upon complete hair regrowth rather than at the first signs of regrowth. The Brazilian Expert Consensus most frequently employs diphencyprone (DPCP), prepared in acetone and stored in amber bottles, with treatment structured into three phases: initiation, follow-up, and maintenance. The initiation phase involves applying 2% DPCP to an inconspicuous site, such as behind the ear, while avoiding sun exposure and washing for 48 h. The follow-up phase begins a few weeks later with weekly applications of progressively lower concentrations than the initial phase, titrated to induce mild to moderate skin reactions. Once an effective concentration is reached, it is maintained weekly. After achieving cosmetically acceptable hair regrowth, the application frequency is gradually reduced to every two weeks, then monthly, before eventual discontinuation. If no response is observed after six months, alternative therapies may be considered; although in some cases, treatment may need to be continued for up to two years to achieve regrowth. Therapy is maintained until satisfactory hair recovery is achieved.

Similarly, Saudi experts recommend DPCP, but also suggest squaric acid dibutyl ester (SADBE) if prior treatment proves ineffective. They strongly advise against abrupt discontinuation, due to the risk of relapse. The Australian Expert Consensus proposes topical or systemic immunotherapy for patients with stable but extensive disease, those unresponsive to intralesional therapy, or those experiencing adverse effects from topical or intralesional corticosteroids, and additionally notes its potential use in promoting regrowth in patients with limited AA. Notably, contact immunotherapy is not recommended in either the European consensus or the ACE consensus [3,4,5,6,7].

### 7.3. Anthralin

Anthralin is widely prescribed by dermatologists for limited patchy AA and is considered a safe and viable treatment option with minimal systemic toxicity; nevertheless, the ACE study does not indicate its use. Similarly, its use is not indicated in the expert consensus from Australia nor Europe. On the contrary, the expert consensus from Brazil and Saudi Arabia suggest applying the cream for 30 min and then increasing the time every three days by 15 min up to a maximum of 2 h [3,4,5,6,7].

### 7.4. Minoxidil

The linear growth rate of hair regrowing within a patch of AA seems to be accelerated with the use of topical minoxidil; nonetheless, not all alopecia patients require its use. The ACE study suggested that topical minoxidil can be administered in combination with other topical or systemic agents; however, its efficacy has been questioned. In a comparative study by El Taieb et al., 5% topical minoxidil was found to be less effective than platelet-rich plasma, with the latter producing faster hair regrowth and reducing both short vellus and dystrophic hairs. Low-dose oral minoxidil has also been explored as an adjuvant option, though current evidence remains limited and does not support its use as monotherapy. Limited research indicates that the combination of oral minoxidil and tofacitinib may be more effective than tofacitinib alone. Comparable results were observed in meta-analysis when the 308 nm excimer laser/light or He-Ne laser therapy were used as adjuncts [4,69,70,71,72].

### 7.5. Steroid-Sparing Agents: Azathioprine, Methotrexate, Cyclosporine and Sulfasalazine

Regarding steroid-sparing agents, the most commonly used in clinical practice are azathioprine, methotrexate, cyclosporine, and sulfasalazine. The ACE study does not recommend the use of azathioprine, citing insufficient supporting evidence in the current literature. Methotrexate, however, may be used as monotherapy in severe cases of AA, with recommended doses of 15–20 mg weekly in adults and 0.4 mg/kg/week in adolescents aged 13–18 years. The Saudi Expert Consensus similarly recommends a weekly dose of 15–20 mg, whereas the European Expert Consensus suggests a range of 15–25 mg/week. The Australian Expert Consensus advises starting with 5–10 mg/week and titrating every 4–6 weeks up to 20–30 mg/week. The Brazilian guidelines also recommend gradual titration, beginning with 5–10 mg/week and increasing to 20–25 mg/week as tolerated [3,4,5,6,7].

Cyclosporine is classified as an effective monotherapy in the ACE study, with a recommended dose of 5 mg/kg/day. The study emphasizes the importance of not exceeding this dosage, as lower doses have also demonstrated efficacy. Cyclosporine has been shown to be effective both as monotherapy and in combination with glucocorticosteroids (GCS). Notably, a meta-analysis reported higher response rates with combination therapy (69%) compared to monotherapy (57%), along with a lower relapse rate (36% vs. 74%, respectively). The European Expert Consensus recommends the same dosing strategy as the ACE study. In contrast, the Brazilian and Australian guidelines suggest starting at 2 mg/kg/day divided into three doses, with titration every 4–6 weeks up to a maximum of 5 mg/kg/day, depending on patient tolerance. Saudi experts recommend 3–5 mg/kg/day. The use of sulfasalazine is not endorsed by the ACE study or by the Australian, European, or Brazilian expert consensuses. However, the Saudi Expert Consensus suggests initiating therapy with 0.5 g twice daily for one month, increasing to 1 g twice daily for the following month, and finally to 1.5 g twice daily for four months [3,4,5,6,7].

### 7.6. New Emerging Therapies

The landscape of treatments for AA is broad and the potential benefits and harms of available treatments are not widely discussed and practiced. As a result of this, Cochrane Library recently published a network meta-analysis of the treatments for AA with the aim of assessing the benefits and harm of the existing treatments [73,74]. The authors reviewed randomized controlled trials (RCTs) conducted up to July 2022 that evaluated classical immunosuppressants, biologics, small-molecule inhibitors, contact immunotherapy, and hair growth stimulants. The conclusions of the meta-analysis were largely disappointing, with only one promising finding: baricitinib was shown to significantly increase the rate of >75% hair regrowth compared with the placebo. The authors further noted that the evidence regarding the effect of oral cyclosporine on health-related quality of life remains inconclusive. Overall, the level of confidence in the remaining evidence was considered low, primarily due to the limited sample sizes and methodological weaknesses of the available studies.

#### 7.6.1. Janus Kinase (JAK) Inhibitors

JAK inhibitors have now emerged as a novel and promising class of drug for treating severe AA which is highly encouraging, especially since AA is a historically challenging-to-treat condition. Baricitnib was the first JAK inhibitor that became FDA-approved in treating severe AA; however, the drug has now been accompanied by several other promising agents from the same drug group [75]. Recently, the FDA approved ritlecitnib for individuals with severe AA who are 12 years of age or older (Pfizer), unlike baricitinib, which is approved for treatment of severe AA in adults > 18 years of age [76,77]. As of July 2024, deuroxolitinib (formerly CTP-543) has also been approved by the FDA for the treatment of severe AA. Other agents that remain unapproved by the FDA but are frequently used off-label in clinical practice include tofacitinib, ruxolitinib, upadacitinib, delgocitinib, and brepocitinib. Both the European and Saudi Expert Consensuses recommend tofacitinib at a dose of 5 mg twice daily and ruxolitinib at a dose of 20 mg twice daily. In addition, the European Expert Consensus supports the off-label use of upadacitinib at 30 mg once daily and notes the use of delgocitinib in Japan at the same dosage [3,4,5,6,7].

The Summary of Product Characteristics (SmPC) recommends baricitinib at a daily dose of 4 mg; however, a reduced dose of 2 mg once daily may be considered for patients over 75 years of age or those with a history of chronic or recurrent infections. A dose reduction from 4 mg to 2 mg is also advisable in patients who have achieved adequate disease control. The EMA safety precautions for JAK inhibitors must be carefully observed when prescribing baricitinib. For ritlecitinib, the EMA-approved dose is 50 mg once daily. In comparison, the Saudi Expert Consensus recommends either 30 mg or 50 mg daily. Laboratory monitoring should include platelet and lymphocyte counts, as specified in the SmPC [68].

In 2023, the EMA recommended that JAK inhibitors (tofacitinib, baricitinib, upadacitinib, abrocitinib, filgotinib) should only be used in patients without suitable alternative treatment options, specifically those over 65 years of age, individuals at increased risk of cardiovascular events, smokers, or patients at increased risk of cancer. Additional caution is advised in patients with known risk factors for venous thromboembolism. This recommendation was based on the results of the phase 3b/4 ORAL Surveillance trial, conducted in patients with rheumatoid arthritis aged >50 years with at least one cardiovascular risk factor, which demonstrated that adverse events were more frequent with tofacitinib compared to TNF inhibitors such as adalimumab or etanercept. It is worth noting, however, that some experts have questioned these recommendations, emphasizing that they were derived from a single study, in one disease population, and in comparison with TNF inhibitors, which are themselves considered cardioprotective. Nevertheless, recent systematic reviews have confirmed that the safety profile of JAK inhibitors in AA is generally favorable in both adults and children, with the most commonly reported adverse effects being headache, acne, and an increased risk of upper respiratory tract infections [3,78].

Husein-ElAhmed conducted a systematic review where the authors investigated the comparative efficacy of oral JAK inhibitors and biologics in adult AA. The assessment of the efficacy was based upon two outcomes; SALT50 and the mean change in SALT from baseline [79,80]. The SALT50 outcome is considered an acceptable endpoint for trials involving extensive AA and systemic agents. Ivarmacitinib 4 mg and ritlecitinib 200/50 mg both showed noteworthy efficacy and ranked high in both outcomes; however, baricitinib 4 mg was the agent with the highest probability of being the most effective [80,81]. Compared with other JAK inhibitors, baricitinib 4 mg and ritlecitinib 200/50 mg may offer unique therapeutic advantages, as both demonstrated a clear dose-dependent effect, which has not been observed with ivarmacitinib. Accordingly, JAK1- or JAK3-selective inhibitors appear to be the most suitable options for AA, owing to their higher efficacy and lower risk of hematologic toxicity. The authors further noted that targeting the Th2 axis with agents such as dupilumab or tralokinumab yielded less favorable outcomes than most JAK inhibitors. Similarly, therapies targeting phosphodiesterase 4, interleukin-17, or employing low-dose interleukin-2 to enhance Treg homeostasis have also shown limited benefit in managing AA [80].

#### 7.6.2. Biologicals

The use of biologic agents is not recommended in the Australian, Brazilian, European, or Saudi consensuses. Nevertheless, biologics have attracted growing interest in the treatment of alopecia areata, providing potential new therapeutic options. Among them, dupilumab has emerged as one of the most extensively studied agents, with several studies suggesting a beneficial effect on hair regrowth [82,83,84,85]. Nonetheless, some reports also highlight the potential for AA onset or relapse during or after treatment with dupilumab, underscoring the complexity of its role in AA management [55,86].

In clinical trials, abatacept has shown potential as a treatment for AA. In a study of 15 patients, 9 demonstrated varying degrees of positive response, including 1 patient who achieved complete scalp hair regrowth by week 36. Based on these findings, Mackay-Wiggan et al. recommend further investigation of abatacept, particularly in combination therapy approaches [87].

Similarly, ustekinumab has demonstrated encouraging results. Guttman-Yassky et al. reported hair regrowth in all 3 patients with moderate to severe AA who were treated with the biologic, further supporting its potential as an effective therapeutic option for AA [88]. It is important to note that ustekinumab has also been associated with the induction of AA in some cases [89,90]. Expert consensus indicates that ustekinumab is ineffective when combined with systemic steroids. Similarly, other biologic agents—including etanercept, adalimumab, infliximab, and alefacept—have demonstrated limited or no efficacy in the treatment of AA, underscoring the need for further research into more targeted therapeutic strategies for this challenging condition [91].

#### 7.6.3. Small-Molecule Inhibitors

The Australian consensus reports multiple cases of hair regrowth following treatment with tofacitinib, as well as near-complete regrowth in three patients treated with ruxolitinib for myelofibrosis. The Brazilian consensus also highlights tofacitinib and ruxolitinib as effective options for patients unresponsive to other therapies. According to the Saudi consensus, tofacitinib 5 mg twice daily may be combined with systemic steroids in cases of treatment-resistant AA. Furthermore, combining tofacitinib 5 mg twice daily with low-dose oral minoxidil (2.5 mg once daily for women and 2.5 mg twice daily for men) has shown encouraging results with minimal adverse effects. The use of small-molecule inhibitors is not addressed in the Experts Consensus.

Recently, small-molecule inhibitors have gained attention as promising therapeutic options for AA. In a study by Estébanez et al. investigating apremilast in patients with refractory AA, treatment resulted in complete hair regrowth in one patient and significant improvement in others, suggesting potential efficacy of this PDE4 inhibitor in cases resistant to conventional therapies [92]. However, in a randomized, placebo-controlled, single-center pilot study by Mikhaylov et al., participants did not achieve clinically significant hair regrowth compared with the placebo group, suggesting that although apremilast may hold some therapeutic potential, it is likely insufficient for moderate-to-severe cases of AA [93].

However, according to the most recent Cochrane review, both ruxolitinib and tofacitinib showed very uncertain results with minimal evidence of significant hair regrowth [74].

#### 7.6.4. Exosomes

One promising development in hair loss therapy is the use of exosomes, which have attracted growing interest among researchers and clinicians for their potential diagnostic and therapeutic applications. Esther Lee et al. reported on the potential of adipose stem cell-derived exosomes in treating hair loss, demonstrating their beneficial effects on hair biology by promoting cell proliferation, elongation of hair follicles, and upregulation of gene expression associated with hair growth [94]. The initial clinical findings were encouraging; however, as of 2024, there are currently no exosome-based hair loss treatments [95].

#### 7.6.5. Excimer Laser

Although excimer light was not formally indicated as a therapeutic option in Australian, European or Experts consensus, the Saudi experts briefly acknowledged its potential benefits as an alternative treatment. In contrast, the Brazilian consensus explicitly cites several studies demonstrating its efficacy in inducing hair regrowth in patients with alopecia areata, while also highlighting the relatively high cost of this technology as a limiting factor for broader use.

#### 7.6.6. UVB Phototherapy

Despite not being mentioned in any of the consensuses, UVB therapy showed promise in a retrospective review by Salman et al. Using targeted NB-UVB, they treated 173 patients with a variety of dermatoses, including 32 cases of AA. Most patients (88.2%) had previously failed topical therapies. Clinical assessment revealed that 40.6% of patients achieved moderate or greater improvement, defined as more than 50% hair regrowth, while 28.1% attained complete response with over 90% regrowth. Excluding cases lost to follow-up, the complete response rate increased to 52.9%. Mild erythema was reported in just over half of the patients, whereas severe adverse events were rare [96]. 

Limitations and side effects of each agents are indicated in Table 2.

## 8. Prognosis

AA has a highly unpredictable course; however, spontaneous recovery of hair growth is seen in 34–50% within one year after AA occurrence [119]. Descriptions of the poor prognostic factors associated with the disease’s progression dominate in the literature compared to factors contributing to the remission of the disease. The prognostic factors of the disease that are commonly associated with relapse are described thoroughly in the literature; the same applies for the clinical features reported to increase disease severity, as stated in Table 3.

## 9. Conclusions

International guidelines highlight individualized treatment, but translation into practice should consider age, comorbidities, and psychological burden. Personalized therapy especially in children, patients with autoimmune comorbidities, and those with significant psychosocial distress represents a critical step forward. Future research should focus on long-term safety and efficacy of JAK inhibitors and other novel agents, identification of biomarkers to guide therapy, clinical translation of exosome- and stem-cell-based therapies, and integration of psychosocial outcomes as core endpoints. In conclusion, substantial therapeutic progress has reshaped the AA landscape, yet bridging innovation with clinical translation remains essential. A biomarker-driven, multidisciplinary approach will be pivotal to achieving true precision medicine and improving long-term outcomes in alopecia areata.

## Figures and Tables

**Table 1:** Co-morbidities and triggers of the disease.

Most common co-morbidities: thyroid disease, diabetes mellitus, inflammatory bowel disease, systemic lupus erythematosus, rheumatoid arthritis, psoriasis with psoriatic arthritis, allergic rhinitis, asthma, eczema, contact dermatitis, depression, anxiety. Malignancies: thyroid cancer, myasthenia gravis and thymoma, primary cutaneous follicle center lymphoma, Hodgkin’s lymphoma [36,37,38,39]. Cardiovascular diseases: acute myocardial infarction [40].
Triggers: anxiety, smoking, sleeping disorders, stress [8,41,42,43,44]. Infections: Epstein–Barr infectious mononucleosis, human immunodeficiency virus (HIV) infection, Human Papillomavirus Infection [45,46,47]. Vaccinations: herpes zoster, hepatitis B, Japanese encephalitis, influenza, quadrivalent HPV, COVID-19 [48,49,50,51]. Drugs: Adalimumab, Denosumab, Dupilumab, Etanercept, Infliximab, Nivolumab, Omalizumab, Pembrolizumab, Secukinumab [52,53,54,55,56,57,58,59,60,61]. Other: vitamin D deficiency, decreased serum ferritin, low serum zinc [62,63,64,65,66].

**Table 2:** Limitations and side effects of each agent.

Group of Drugs	Agent	Limitations	Adverse Side Effects
Glucocorticosteroids (GCS) [4,5,8,97,98,99,100,101,102,103,104]	Intralesional GCS	Require multiple treatments and cause patient discomfort and pain	Possible systemic effects Dyspigmentation Skin atrophy Anaphylaxis (rarely)
	Topical GCS	Limited benefit in patchy AA++ Patients commonly suffer relapses Ultrapoent GCS, e.g., clobetasole, should be avoided in the eyebrow area	Folliculitis Local skin atrophy Striae Acne Teleangiectasia Dyschromia Adrenal suppression (rarely)
	Systemic GCS	Not favored for chronic AA due to long-term therapy safety concerns that are associated with prolonged therapy	Weight gain Cushing’s disease Hypertension Osteoporosis Acne
Contact immunotherapy [103,105,106,107]	DPCP SADBE	Immunotherapy is an absolute contraindication due to opposing mechanism of action Desensitization and allergic reactions may occur in the clinicians who apply the preparation to patient’s scalp	Contact eczema Urticaria Lymphadenopathy
	Anthralin	High relapse rate (up to 64%) Low effectiveness limits its use in clinical practice Based on the available data, the estimated efficacy is 32–33% of partial hair regrowth with the maximum effect occurring 9–15 months of therapy	Skin irritation Lymphadenopathy
Hair growth stimulants [4,70,103,108,109,110,111]	Topical Minoxidil	Its efficacy as adjuvant drug is still under investigation, with some data indicating that it could potentially accelerate hair regrowth in hairless patches; however, numerous data have proven that it is ineffective as monotherapy Some clinicians recommend regular monitoring of blood pressure, heart rate and electrocardiographic changes, fundoscopic examination and renal function	Hypertrichosis Contact dermatitis Transient shedding Sparse vellus hair on various body parts Tachycardia
	Systemic Minoxidil	Limited data on its efficacy in inducing hair regrowth; hence, it should not be used in monotherapy	Lightheadedness Fluid retention Tachycardia Headache Periorbital edema Insomnia
Prostaglandin analogs [3,5,8,103]	Latanoprost Bimatoprost	Latanoprost has been associated with irreversible iridial pigmentation	Transient mild eye irritation Hyperemia Conjunctivitis
Steroid sparing agents [3,5,10,103,112]	Azathioprine	The literature data are insufficient to recommend this agent Thiopurine methyltransferase (TPMT) activity should be monitored prior to treatment and the dose should be modified according to TPMT activity	Gastrointestinal upset Altered thiopurine methyltransferase (TPMT) activity Elevated liver enzymes Pancreatitis Bone marrow suppression
	Methotrexate	Currently, there are insufficient data to accurately estimate the therapeutic benefit of methotrexate as a monotherapy Its efficacy ranges vary widely in the literature data with 2.2–50% of patients achieving a therapeutic response. Better documented is the efficacy of methotrexate used in combination with GCS	Nausea Leukopenia Transient elevation of hepatic enzymes Teratogenic Nephrotoxic Hepatotoxic
	Ciclosporin	The high rates of recurrence after discontinuation of the drug and its adverse side effects limits its use	Nephrotoxicity Immunosuppression Arterial hypertension
Miscellaneous [113,114]	Sulfasalazine	Close follow-up consisting of monitoring G6PD, blood count, biochemistry and hepatogram assesmnet is essential Discontinuation of the drug have displayed high occurrence of relapses	GI-tract distress Rashes Headaches Laboratory anomalies
Complementary and alternative medicine (CAM) [97,103,115,116,117,118]	Photochemotherapy. Psoralen plus ultraviolet A (PUVA)	Continued therapy is needed to maintain hair growth, which may lead to unacceptable high cumulative UVA dose High relapse rate and low response rate	Acute phototoxic reactions Hyperpigmentation Increased risk for long-term skin damage, e.g., photoaging Increased risk of skin cancer with prolonged PUVA use Nausea and gastrointestinal discomfort from oral psoralen
	Laser therapy	Can be applied as an alternative therapy only in selected cases and as an adjuvant therapy in acutely resistant AA patches High cost with limited efficacy	Mild erythema Contact eczema Blistering Pruritus Hyperpigmentation Mild peeling of skin
	Aromatherapy	Results of the studies must be interpreted with caution given small study size and variable quality of study design. Disease length and severity for participants studied is unknown; consequently, generalizability to patients with varied degrees and duration of hair loss remains unclear.	Irritation at the application site Essential oils influence the skin barrier function and may induce contact dermatitis
	Hypnotherapy	Non-randomized trial failed to show any difference in hair growth in the treated group compared to the placebo group Effectiveness remains unclear due to small study samples in the current evidence	Headache Dizziness Anxiety Creation of false memories

**Table 3:** Poor prognostic factors commonly associated with disease relapse and clinical features reported to increase disease severity.

Clinical Characteristics of Poor Prognostic Factors
Body hair involvement [12,120] Young age at disease onset [5,120,121,122] Extensive hair loss [5,12,120,123] Ophiasis pattern of hair loss [5,124] Family history [5,8,108,125] Severity of AA at first consultation [6] Concurrent autoimmune or atopic disease [5,122] Thyroid disease [122] Nail findings [5,126,127] Smoking [41] Episode duration longer than 1 year [5] Genetic disease association, for example, Down syndrome [5]
**Clinical Features Reported to Increase Disease Severity**
Extensive hair loss [12] Concomitant autoimmune disease [12] Atopic dermatitis [127] Nail findings [126]
